# Chromosomal Evolution in Tortricid Moths: Conserved Karyotypes with Diverged Features

**DOI:** 10.1371/journal.pone.0064520

**Published:** 2013-05-24

**Authors:** Jindra Šíchová, Petr Nguyen, Martina Dalíková, František Marec

**Affiliations:** 1 Institute of Entomology, Biology Centre ASCR, České Budějovice, Czech Republic; 2 Faculty of Science, University of South Bohemia, České Budějovice, Czech Republic; Natural Resources Canada, Canada

## Abstract

Moths of the family Tortricidae constitute one of the major microlepidopteran groups in terms of species richness and economic importance. Yet, despite their overall significance, our knowledge of their genome organization is very limited. In order to understand karyotype evolution in the family Tortricidae, we performed detailed cytogenetic analysis of *Grapholita molesta*, *G. funebrana*, *Lobesia botrana*, and *Eupoecilia ambiguella*, representatives of two main tortricid subfamilies, Olethreutinae and Tortricinae. Besides standard cytogenetic methods, we used fluorescence *in situ* hybridization for mapping of major rRNA and histone gene clusters and comparative genomic hybridization to determine the level of molecular differentiation of the W and Z sex chromosomes. Our results in combination with available data in the codling moth, *Cydia pomonella*, and other tortricids allow us a comprehensive reconstruction of chromosomal evolution across the family Tortricidae. The emerging picture is that the karyotype of a common ancestor of Tortricinae and Olethreutinae differentiated from the ancestral lepidopteran chromosome print of n = 31 by a sex chromosome-autosome fusion. This rearrangement resulted in a large neo-sex chromosome pair and a karyotype with n = 30 conserved in most Tortricinae species, which was further reduced to n = 28 observed in Olethreutinae. Comparison of the tortricid neo-W chromosomes showed differences in their structure and composition presumably reflecting stochasticity of molecular degeneration of the autosomal part of the neo-W chromosome. Our analysis also revealed conservative pattern of the histone distribution, which is in contrast with high rDNA mobility. Despite the dynamic evolution of rDNA, we can infer a single NOR-chromosome pair as an ancestral state not only in tortricids but probably in all Lepidoptera. The results greatly expand our knowledge of the genome architecture in tortricids, but also contribute to the understanding of chromosomal evolution in Lepidoptera in general.

## Introduction

Moths and butterflies (Lepidoptera) constitute, with nearly 160,000 described species, one of the largest groups of animals [1]. Despite the species richness, the Lepidoptera are far more homogeneous, structurally and ecologically, than the other large insect orders such as Coleoptera, Diptera, and Hymenoptera [2]. This also applies to their cytogenetic characteristics. Holokinetic chromosomes of Lepidoptera possess very few differentiating features. They lack primary constrictions (centromeres *sensu stricto* [3]) and they are usually small, numerous, and uniform in shape. Although their holokinetic structure is expected to facilitate karyotype evolution via chromosome fusion and fission (see discussion in [4]), the architecture of lepidopteran genomes appears to be relatively stable. Most species have haploid chromosome numbers close to 30, and the modal number of n = 31 occurs from basal to advanced clades [5]–[8]. Recent comparative genomic studies revealed extensive conserved synteny of genes between the silkworm (*Bombyx mori*) and several other lepidopteran species (e.g. [4], [9]–[13]), which suggests evolutionary stability of whole genomic regions. Additionally, these studies established the chromosome number of n = 31 as an ancestral karyotype of non-tineoid Ditrysia (*sensu* [14]). The high degree of conservation at the chromosomal level across the phylogenetic tree of Lepidoptera contrasts with exceptional diversity found in some taxa [15], [16].

The family Tortricidae with about 10,300 described species of moths includes almost 700 potential pests of agricultural, forest, and ornamental plants [17]–[19], and it is thus among major lineages of basal Ditrysia in terms of species richness and economic importance. The family is comprised of three subfamilies, Chlidanotinae, Tortricinae, and Olethreutinae [20]. A recently published molecular analysis of phylogenetic relationships within tortricids confirmed Chlidanotinae as the earliest diverging lineage and supported Tortricinae and Olethreutinae as sister groups [21]. The overall significance of tortricids is demonstrated by numerous studies on various aspects of their taxonomy, biology, and pest control [22]. However, cytogenetics of tortricids is poorly explored. Nothing is known about chromosomes in Chlidanotinae, the smallest subfamily with about 240 species [20]. In the other two subfamilies, published cytogenetic data are available for 40 species, mostly reporting only chromosome numbers in males. In Tortricinae, 24 out of 25 species examined have the same haploid chromosome number of n = 30 [5], [23]–[28]. These include, for example, the spruce budworm *Choristoneura fumiferana* (Clemens), which is one of the most destructive pests of coniferous forests in North America. In Olethreutinae, eight out of 15 species examined have n = 28; other species have different, mostly reduced chromosome numbers [25], [26], [29]–[32].

The only tortricid in which detailed cytogenetic research was performed is the codling moth, *Cydia pomonella* (L.). This species belongs to the tribe Grapholitini of the subfamily Olethreutinae, and its larva is a well-known pest of pome fruits (apple, pear, and quince) and walnuts [33]. The codling moth has n = 28 and a WZ/ZZ (female/male) sex chromosome system [32]. In contrast to a typical lepidopteran karyotype, which shows a gradual decrease in chromosome size (e.g. [34]; reviewed in [7]), the codling moth karyotype consists of chromosomes of several size-groups and has two special features: (i) although it has two nucleolar organizer regions (NORs) as several other lepidopteran species, the NORs are located at the opposite ends of a single autosome [32], [35]; (ii) both the W and Z sex chromosomes are remarkably larger than the autosomes [32], which is unusual in the Lepidoptera (cf. [4], [34], [36], [37]). Nevertheless, basic characteristics of the codling moth sex chromosomes are similar to those found in other lepidopterans (reviewed in [38], [39]). The W and Z chromosome, though similar in size, are highly differentiated from each other. The Z chromosome is composed of gene-rich euchromatin and resembles the autosomes. In contrast, the W chromosome is heterochromatic and composed mainly of repetitive DNA sequences [32], [40].

Recently, we have physically mapped the large Z chromosome of the codling moth using fluorescence *in situ* hybridization (FISH) with bacterial artificial chromosome (BAC) probes, the so-called BAC-FISH, and showed that it is in fact a neo-Z chromosome that has arisen by fusion between an ancestral Z chromosome and an autosome corresponding to chromosome 15 in the *Bombyx mori* reference genome. Further experiments, performed by quantitative PCR (qPCR) showed a Z-linkage of selected orthologs of *B. mori* chromosome 15 genes in two other tortricids, *Lobesia botrana* and *Eupoecilia ambiguella* (see below). The results suggest that the Z chromosome-autosome fusion originated in a common ancestor of the main tortricid subfamilies, Olethreutinae and Tortricinae [41].

In this study, we examined karyotype features in four other species of tortricids by standard cytogenetic techniques and by mapping multigene families (major rRNA genes and histone genes) using fluorescence *in situ* hybridization (FISH) with 18S rDNA and H3 histone probes. We also used comparative genomic hybridization (CGH) to determine the level of molecular differentiation of the W and Z sex chromosomes. Cytogenetic characteristics were compared with those of the codling moth [32] with the aim to understand karyotype and sex chromosome evolution in the family Tortricidae. Such complex comparisons have never been done across any lepidopteran family, except for the W chromosome divergence in the family Pyralidae [37]. For our research we chose two pests of pome and stone fruits, the Oriental fruit moth, *Grapholita molesta* (Busck) and the plum fruit moth, *Grapholita funebrana* (Treitschke), both close relatives of the codling moth (Olethreutinae: Grapholitini), and two pests of cultivated grapes, the European grapevine moth, *Lobesia botrana* (Denis & Schiffermüller) from the tribe Olethreutini and the vine moth, *Eupoecilia ambiguella* (Hübner) representing the tribe Cochylini of Tortricinae. Our choice was also motivated by the fact that *G. molesta* and possibly the two grape pests are candidate species for their control by sterile insect technique (SIT), which is currently used against the codling moth [42], and the acquired cytogenetic knowledge may facilitate transfer of the technology to these and other tortricid pests.

## Materials and Methods

### Insects

We used a laboratory wild-type strain of the codling moth, *Cydia pomonella*, referred to as Krym-61 (for its origin, diet and rearing conditions, see [32]). A laboratory culture of *Grapholita molesta* was obtained from Beatrice Christoffel and Silvia Dorn (Applied Entomology, Institute of Agricultural Sciences, ETH Zürich, Switzerland). The culture was established from a wild population collected in orchards in the province Emilia Romagna, Italy (see [43]). For *G. molesta*, we used the same diet and rearing conditions as for the codling moth. Laboratory cultures of *Lobesia botrana* and *Eupoecilia ambiguella*, both originating from field collections in wine-growing regions in Germany, along with a rearing protocol and composition of artificial diet were obtained from Annette Reineke (Department of Phytomedicine, Research Center Geisenheim, Germany). The diet was prepared according to the recipe of Christoph Hoffmann (Julius Kühn Institute, Siebeldingen, Germany). Briefly, agar (50 g) was boiled in 1.5 L of water, cooled down to about 60°C, and then supplemented with the following ingredients: wheat germ (187 g), casein (88 g), dried yeast (38 g), Wesson salt mixture (25 g), sugar (74 g), benzoic acid (4 g), cholesterol (2.5 g), methylparaben (2.5 g), ascorbic acid (40 g), Vanderzant vitamin mixture (15 g), chloramphenicol (1 g), formaldehyde (1 mL), and sunflower oil (5 mL). All four tortricid species were reared in a constant-temperature room under non-diapausing conditions (25±1°C; 16 h light : 8 h dark regime), without humidity control.

In *Grapholita funebrana*, it is difficult to establish a laboratory culture. Therefore, we used field-collected larvae from infested plum trees near České Budějovice, along the road between the villages of Zaliny and Ledenice.

This study was performed in strict accordance with the laws of the Czech Republic. Herewith we declare that all species used are agricultural pests not listed as endangered species (see the Decree of the Ministry of the Environment CR no. 395/1992 of the Legal Code, including updated versions), and no permissions are required for their collection and further use for research. The only field-collected species, *G. funebrana*, was sampled in a free-access state land, where no permission is needed.

### Chromosome Preparations

In each species, two types of spread chromosome preparations were made. Meiotic chromosomes were obtained from gonads of the fifth instar male and female larvae as described in [34]. Briefly, after dissection in a physiological solution testes were pretreated for 10 min in a hypotonic solution (0.075 M KCl), fixed in Carnoy fixative (ethanol/chloroform/acetic acid, 6∶3:1) for 15 minutes, dissociated with tungsten needles in a drop of 60% acetic acid and spread on the slide using a heating plate at 45°C. Ovaries were fixed without hypotonization to preserve the chromomere pattern of pachytene bivalents. Mitotic chromosomes were obtained from wing imaginal discs of the fifth instar larvae of both sexes. Wing discs were dissected out in a physiological solution, swollen for 20 min in 0.075 M KCl, and further processed as described above.

For visualization of the W chromosome, spread pachytene oocytes were stained in 2.5% lactic acetic orcein and inspected with phase-contrast optics. This technique is routinely used in Lepidoptera as it often allows identification of the sex chromosome bivalent by densely stained heterochromatin of the W chromosome, while autosome bivalents and the Z chromosome show a chromomere-interchromomere pattern [38]. For chromosome counts, preparations from wing discs were directly stained with 0.5 µg/mL DAPI (4′,6-diamidino-2-phenylindole; Sigma-Aldrich, St. Louis, MO, USA) in antifade based on DABCO (1,4-diazabicyclo[2.2.2]octane; Sigma-Aldrich). Preparations for FISH techniques were passed through a graded ethanol series (70%, 80%, and 100%, 1 min each) and stored at −80°C until further use.

### Preparation of Polyploid Nuclei

Malpighian tubules from fifth instar male larvae, third and fifth instar female larvae, and adult females were dissected out in a physiological solution, briefly fixed in Carnoy fixative and stained in 1.5% lactic acetic orcein. Preparations were inspected in a light microscope for the presence of female specific sex chromatin (see [44]).

### FISH with 18S rDNA and H3 Histone Probes

Unlabeled 1650 bp long 18S rDNA probe was generated by PCR from the codling moth genomic DNA (gDNA) extracted from adults by standard phenol-chloroform extraction as described in [32]. The probe was labeled with biotin-16-dUTP (Roche Diagnostics GmbH, Mannheim, Germany) by nick translation using Nick Translation Kit (Abbott Molecular Inc., Des Plaines, IL, USA).

To prepare a H3 histone probe specific to codling moth, we used two degenerate primers, forward (5′-ATGGCNCGTACNAARCARAC-3′) and reverse (5′-TANGCACGYTCNCGGAT-3′). The primers were designed in conserved regions identified by multiple alignments of H3 amino acid sequences of several insect species. A codling moth orthologous sequence of the H3 histone gene was generated by PCR in an XP Thermal Cycler (Bioer Technology, Hangzhou, China). Reaction was carried out in 25-µL reaction volumes containing 1× Ex *Taq* buffer, 0.2 mM dNTP mix, 5 µmol each primer, 0.25 U TaKaRa Ex *Taq* Hot Start DNA polymerase (TaKaRa, Otsu, Japan), and about 100 ng of template cDNA prepared from total codling moth RNA as described below. An initial denaturation period of 5 min at 94°C was followed by 30 cycles of 30 s at 94°C, 1 min at 60°C, and 45 s at 72°C, and by a final extension step of 7 min at 72°C. The PCR product showed a single band of about 360 bp on a 1% agarose gel. The band was cut out from the gel, and the DNA was extracted using a Wizard SV Gel and PCR Clean-Up System (Promega, Madison, WI, USA). The extracted DNA sequence was cloned into Promega pGEM T-Easy Vector (Promega), verified by sequencing, and the plasmid was used as a template for PCR amplification of the H3 histone probe. Labeling reaction was carried out in 15-µL volumes containing 1× Ex *Taq* buffer, 0.1 mM of each dATP, dGTP, and dCTP, 0.065 mM dTTP, 0.035 mM biotin-16-dUTP, 5 µmol each M-13 universal primers, 0.25 U TaKaRa Ex *Taq* Hot Start DNA polymerase, and about 5 ng of plasmid DNA. An initial denaturation period of 2 min at 94°C was followed by 30 cycles of 30 s at 94°C, 30 s at 57°C, and 1 min at 72°C, and by a final extension step of 2 min at 72°C.

A total RNA of the codling moth for preparation of the H3 histone probe was isolated from larvae of both sexes by RNA blue (Top-Bio, Prague, Czech Republic) following the supplier’s protocol. RNAs were incubated with DNase I (USB Corporation, Cleveland, OH, USA) for 15 min at 37°C to remove potential contamination by DNAs. The first cDNA strand was synthesized by SuperScript III Reverse Transcriptase (Invitrogen, Carlsbad, CA, USA) following the manufacturer’s protocol; then the reverse transcriptase was inactivated by heating for 15 min at 70°C. Samples were incubated with 5 U RNase H (TaKaRa) for 20 min at 37°C to remove template RNA. RNase H was inactivated by heating for 20 min at 65°C.

FISH with the 18S rDNA and H3 histone probes was carried out as described in [32]. Briefly, chromosome preparations were removed from freezer, dehydrated in the ethanol series, and digested with 100 µg/mL RNase A to remove an excessive amount of rRNAs. After denaturation the chromosomes were hybridized with a probe cocktail containing 15 ng of biotinylated probe and 25 µg of sonicated salmon sperm DNA (Sigma-Aldrich) per slide. Hybridization signals were detected with Cy3-conjugated streptavidin (Jackson ImmunoRes. Labs. Inc., West Grove, PA, USA), amplified with biotinylated anti-streptavidin (Vector Labs. Inc., Burlingame, CA, USA) and again detected with Cy3-conjugated streptavidin. The preparations were counterstained with 0.5 µg/mL DAPI and mounted in antifade based on DABCO.

### Comparative Genomic Hybridization (CGH)

In each species, gDNA was extracted separately from adult males and females by DNeasy Blood & Tissue Kit (Qiagen, Düsseldorf, Germany), except for *G. funebrana* where it was extracted from larvae. Labeling of gDNAs was done using Nick Translation Kit (Abbott Molecular Inc.). Male DNA was labeled with Cy3-dUTP (GE Healthcare, Milwaukee, WI, USA) and female DNA with fluorescein-12-dUTP (Invitrogen). Unlabeled male gDNAs, used as a species-specific competitor, were prepared as follows. In each species, the extracted gDNA was first amplified by GenomiPhi HY DNA Amplification Kit (GE Healthcare) and then sonicated using a Sonopuls HD 2070 (Bandelin Electric, Berlin, Germany), with two cycles of five pulses at 70% power.

CGH was performed according to [36] with several modifications. Briefly, after removal from the freezer, chromosome preparations were dehydrated in the ethanol series, treated with 100 µg/mL RNase A, and denatured. Then the preparations were hybridized with a denatured probe cocktail containing labeled female and male gDNAs (250 ng each), unlabeled sonicated male gDNA (2.5 µg), and sonicated salmon sperm DNA (25 µg) for 3 days at 37°C, washed for 5 min at 62°C in 0.1× SSC containing 1% Triton X-100, counterstained with 0.5 µg/mL DAPI and mounted in antifade based on DABCO.

### Microscopy and Image Processing

Preparations were observed in a Zeiss Axioplan 2 microscope (Carl Zeiss Jena, Germany). Black-and-white images were recorded with a cooled F-View CCD camera and captured with AnalySIS software, version 3.2 (Soft Imaging System GmbH, Münster, Germany). In FISH preparations, images were captured separately for each fluorescent dye and pseudocolored (light blue for DAPI, green for fluorescein, and red for Cy3) and superimposed with Adobe Photoshop, version 7.0.

## Results

### Mitotic Karyotypes

Chromosome numbers were determined from mitotic metaphase chromosomes prepared from wing imaginal discs, which have a high mitotic index in the last (fifth) larval instar because of intensive proliferation of the cells. In each species, several tens of metaphase complements from several specimens of both sexes were examined.

**Figure 1:**
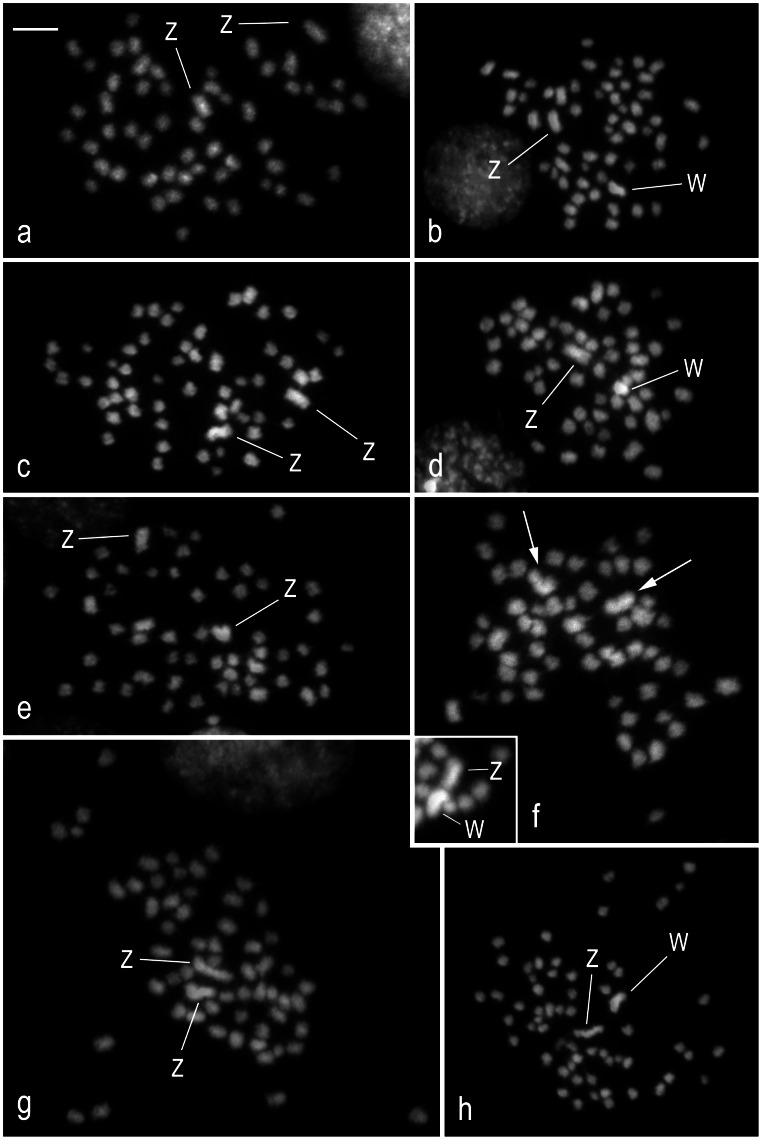
Chromosome preparations of wing discs in four members of the family Tortricidae. Spread mitotic chromosomes were stained with DAPI. White lines point to the largest chromosomes in the karyotype, the W and Z sex chromosomes. *Grapholita molesta* (**a**, **b**): **a** – male mitotic metaphase (2n = 56); **b** – female mitotic metaphase (2n = 56). *Grapholita funebrana* (**c**, **d**): **c** – male mitotic complement (2n = 56); **d** – female mitotic complement (2n = 56). *Lobesia botrana* (**e**, **f**): **e** – male mitotic nucleus (2n = 56); **f** – female mitotic nucleus (2n = 56) with indiscernible sex chromosome pair (arrows); the inset in the bottom left corner shows a detail of another mitotic nucleus with differentiated W and Z chromosomes. *Eupoecilia ambiguella* (**g**, **h**): **g** – spread male mitotic metaphase (2n = 60); **h** – spread female mitotic metaphase (2n = 60). Bar = 5 µm.

Mitotic metaphase complements showed similar features in all tortricids examined (Figure 1a–h). They consisted of mostly rod-shaped chromosomes without any morphological landmarks such as the centromeres, as typical for holokinetic chromosomes in Lepidoptera. There was no difference in chromosome counts between sexes. Based on repeated counts we concluded that three species, *G. molesta* (Figure 1a, b), *G. funebrana* (Figure 1c, d), and *L. botrana* (Figure 1e, f), have identical numbers of 2n = 56 chromosomes like the codling moth, *C. pomonella* (see [32]), whereas the karyotype of *E. ambiguella* consists of a higher chromosome number of 2n = 60 (Figure 1g, h).

In male metaphases of each species, two chromosomes stood out by their large size (Figure 1a, c, e, g). Similarly, two large chromosomes were observed in female metaphases of *G. molesta*, *L. botrana*, and *E. ambiguella* (Figure 1b, f, h). However, in contrast to the largest chromosomes in males they differed from each other by size with the smaller (and/or more compact) chromosome more intensely stained with DAPI. The exception was *G. funebrana*, which showed only one large and one DAPI-highlighted middle-sized chromosome in female metaphases (Figure 1d). Based on the comparison between male and female chromosome complements and also on the analysis of sex chromosome bivalents in pachytene oocytes (see later) we concluded that the two largest chromosomes represent a ZZ pair of the sex chromosomes in males and that the smaller DAPI-positive member of the WZ pair in females is the W chromosome composed of heterochromatin. Very large sex chromosomes including the DAPI-positive W chromosome were previously observed in *C. pomonella* (cf. [32]).

### Chromosomal Location of Major rDNA

**Figure 2:**
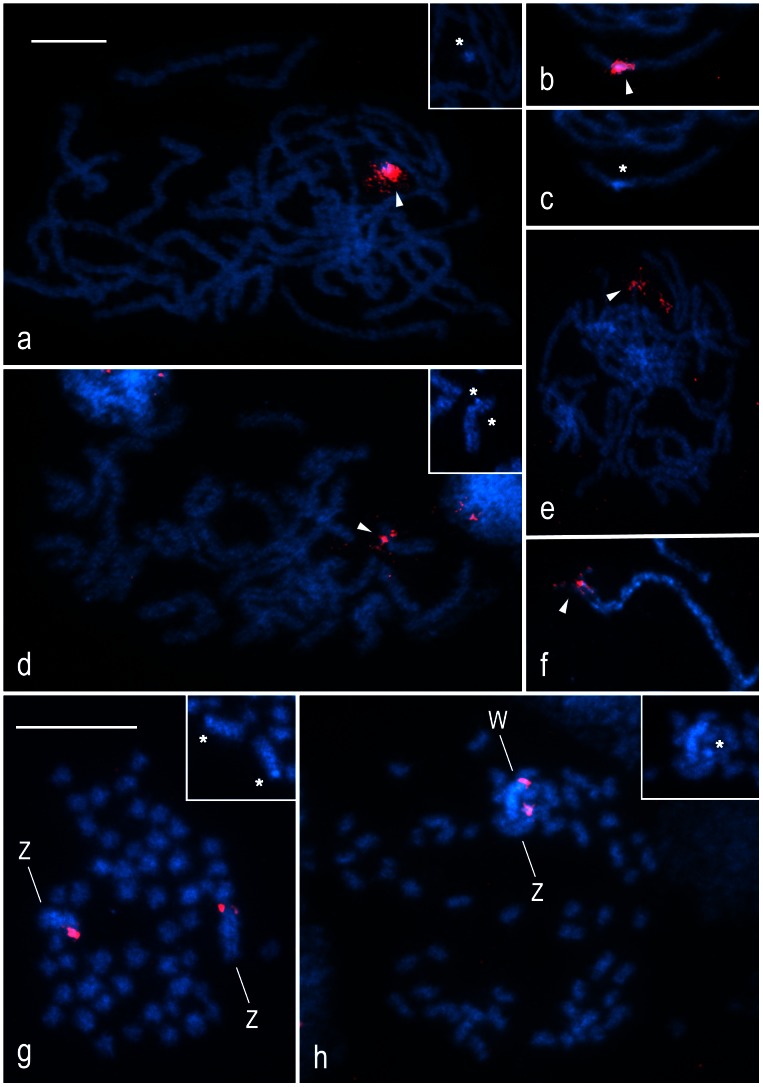
Localization of rDNA clusters in spread chromosome preparations of four species of the family Tortricidae by FISH with 18S rDNA probe. Chromosomes were counterstained with DAPI (blue). Asterisks show DAPI-positive blocks of heterochromatin in the NOR-regions; arrowheads indicate hybridization signals of the 18S rDNA probe (red). *Grapholita molesta*: **a** – male pachytene complement; the inset in the upper right corner shows DAPI image of the NOR-bivalent. *Grapholita funebrana* (**b**, **c**): **b** – composite FISH image of the NOR-bivalent (male pachytene); **c** – DAPI image of the same NOR-bivalent. *Lobesia botrana*: **d** – male pachytene nucleus; the inset in the upper right corner shows DAPI image of the NOR-bivalent. *Eupoecilia ambiguella* (**e**, **f**, **g**, **h**): **e** – male pachytene complement; **f** – a detail of the pachytene WZ bivalent (composite FISH image); **g** – spermatogonial metaphase; **h** – female mitotic metaphase (from wing disc); W and Z indicate sex chromosomes; the insets in the upper right corner of **f** and **g** show DAPI images of the NOR-sex-chromosomes. Bar = 10 µm; **a–f** and **g, h** have the same scale.

The tortricids examined did not differ in the number of rDNA sites. In each species, FISH with the 18S rDNA probe revealed a single cluster of rRNA genes, i.e. one NOR per haploid genome, associated with a small block of DAPI-positive heterochromatin (Figure 2a–h). However, the species differed in the location of rDNA. In pachytene nuclei of *G. molesta*, terminal hybridization signals localized an rDNA cluster to the end of an autosome bivalent (Figure 2a), whereas in the closely related *G. funebrana*, a large interstitial rDNA cluster was found in about one third of an autosome bivalent (Figure 2b, c). In *L. botrana*, an rDNA cluster was positioned near the end of a shorter autosome bivalent (Figure 2d). By contrast, pachytene spermatocytes of *E. ambiguella* showed an rDNA cluster at the end of the longest bivalent (Figure 2e), thus indicating its Z-linkage. Similarly, the probe localized an rDNA cluster to the end of the WZ bivalent in pachytene oocytes (Figure 2f). A comparison of hybridization signals in male and female mitotic metaphases confirmed the terminal location of rDNA in both the W and Z sex chromosomes in *E. ambiguella* (Figure 2g, h) and also confirmed the autosomal location of rDNA in the other species (not shown). Taken together with two rDNA clusters located at the opposite ends of a single autosome pair in the codling moth (see [32]), the results suggest dynamic repositioning of rDNA in tortricids.

### Chromosomal Location of H3 Histone Genes

**Figure 3:**
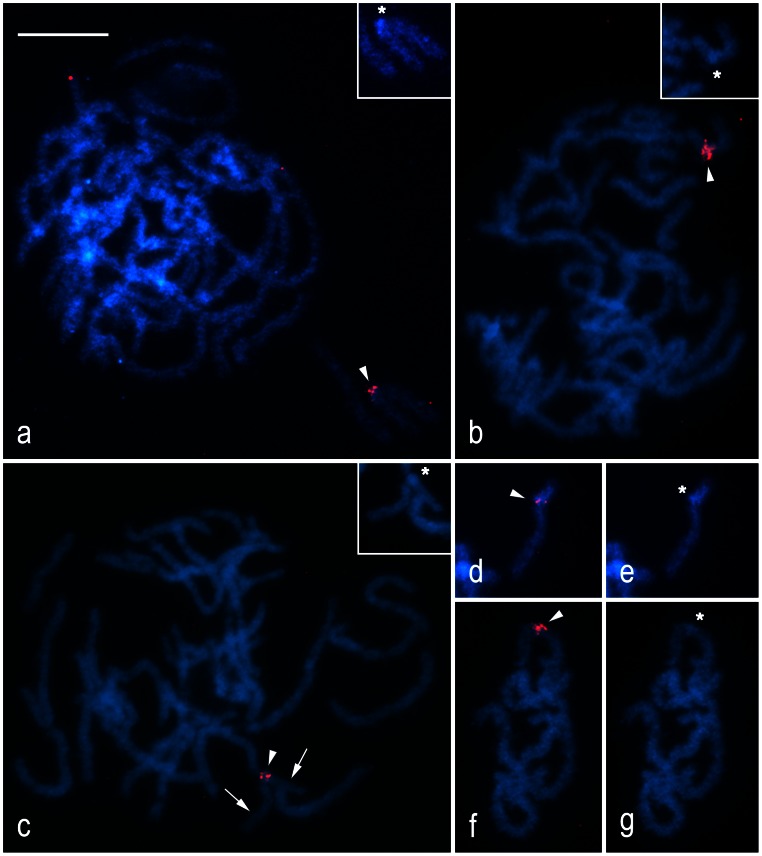
Localization of H3 histone gene clusters in pachytene spermatocytes of five tortricid species by FISH with H3 gene probe. Chromosomes were counterstained with DAPI (blue). Asterisks show DAPI-positive blocks of heterochromatin in the H3-region; arrowheads indicate hybridization signals of the H3 probe (red). *Cydia pomonella*: **a** – pachytene complement; the inset in the upper right corner shows DAPI image of the H3 cluster-carrying bivalent. *Grapholita funebrana*: **b** – pachytene complement; the inset in the upper right corner shows DAPI image of the H3 cluster-carrying bivalent. *Grapholita molesta*: **c** – pachytene complement; the inset in the upper right corner shows DAPI image of the H3 cluster-carrying bivalent. *Lobesia botrana* (**d**, **e**): **d** – composite FISH image of the H3 cluster-carrying bivalent; **e** – DAPI image of the same bivalent. *Eupoecilia ambiguella* (**f**, **g**): **f** – composite FISH image of a part of pachytene nucleus; **g** – DAPI image of the same part of pachytene nucleus. Bar = 10 µm.

FISH with the H3 histone probe was also performed in the codling moth, *C. pomonella*, as it has not been done in this species yet. In pachytene spermatocytes of all five tortricid species, the probe localized a single cluster of H3 histone genes in a shorter autosome bivalent, and similar to rDNA the hybridization signals co-localized with a small block of DAPI-positive heterochromatin (Figure 3a-g). In four species (*C. pomonella*, Figure 3a; *G. funebrana*, Figure 3b; *G. molesta*, Figure 3c; *E. ambiguella*, Figure 3f, g), the hybridization signals positioned the H3 gene cluster near the midpoint of the bivalent, only in *L. botrana* near the end of the bivalent (Figure 3d, e). Similar sizes of the bivalent in all five species as well as similar positions in four of these species suggest a conserved chromosomal location of H3 histone gene cluster in the tortricids.

### Differentiation of Sex Chromosomes

**Figure 4:**
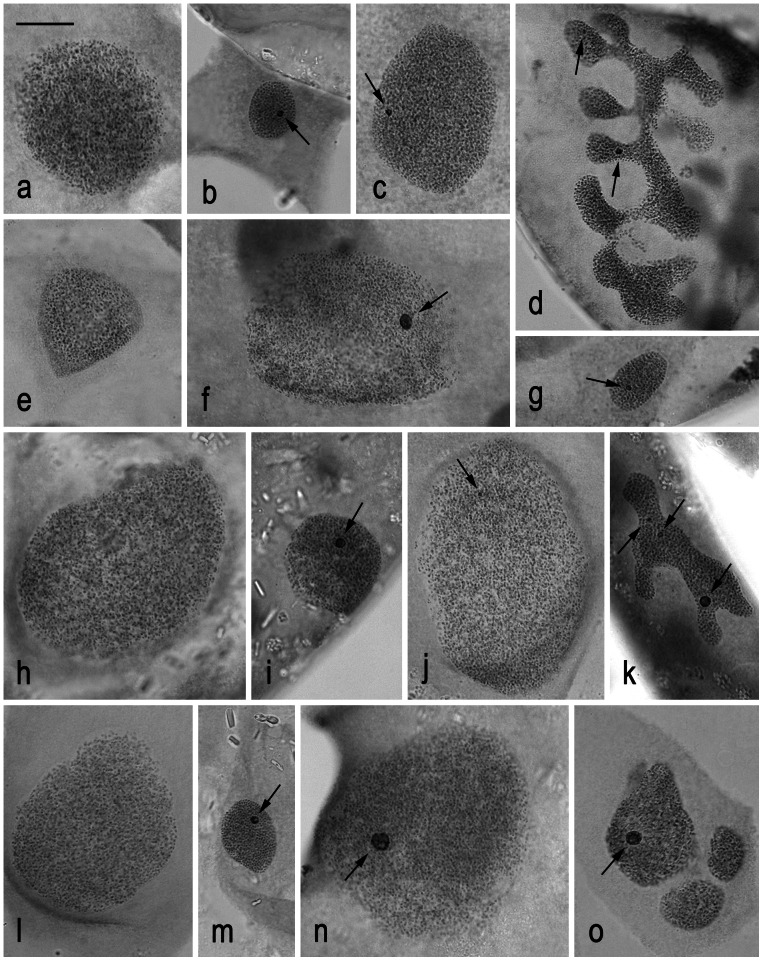
Sex chromatin status in polyploid somatic nuclei of Malpighian tubule cells in four tortricid species. The orcein-stained nuclei were prepared from fifth instar male larvae (**a**, **e**, **h**, **l**), third instar female larvae (**b**, **g**, **i**, **m**), fifth instar female larvae (**c**, **f**, **j**, **n**), and adult females (**d**, **k**, **o**). Arrows indicate deeply stained W chromatin body (-ies). *Grapholita molesta* (**a–d**): **a** – a male nucleus without W chromatin; **b** – a lower-ploidy female nucleus with a relatively large W-body; **c** – a highly polyploid female nucleus with a relatively small W-body; **d** – a branched, highly polyploid female nucleus with W chromatin disintegrated into several small bodies. *Grapholita funebrana* (**e–g**): **e** – a male nucleus without W chromatin; **f** – a highly polyploid female nucleus with a large W-body; **g** – a lower-ploidy female nucleus with a single W-body. *Lobesia botrana* (**h–k**): **h** – a male nucleus without W chromatin; **i** – a lower-ploidy female nucleus with a relatively large W-body; **j** – a highly polyploid female nucleus with a miniature W-body; **k** – a branched, highly polyploid female nucleus with W chromatin disintegrated into several bodies. *Eupoecilia ambiguella* (**l–o**): **l** – a male nucleus without W chromatin; **m** – a lower-ploidy female nucleus with a single W-body; **n** – a highly polyploid female nucleus with a conspicuous W-body; **o** – a fragmented, highly polyploid female nucleus with a conspicuous W-body. Bar = 10 µm.

In each tortricid species, we first examined the status of sex chromatin, which is formed in polyploid somatic nuclei of lepidopteran females by multiple copies of the heterochromatic W chromosome. The sex chromatin is a suitable marker for determining the presence or absence of the W chromosome and also for possible interchromosomal rearrangements involving the W chromosome [38], [44]. As expected, no heterochromatin was observed in somatic polyploid nuclei of males (Figure 4a, e, h, l). In young female larvae of all four species, oval nuclei of a lower ploidy level showed a single heterochromatin body (Figure 4b, g, i, m), indicating the presence of a single W chromosome in the female genomes. However, there were between-species differences in the sex chromatin status of older larvae and adult females. Like in the codling moth (see Figure 4 in [32]), a large single W-body was found in highly polyploid female nuclei of two species, *G. funebrana* (Figure 4f) and *E. ambiguella* (Figure 4n, o). In *G. molesta* larvae, the W-body did not grow proportionally with the nucleus growth (cf. Figure 4b and c). Moreover, the sex chromatin in branched nuclei of adult females was disintegrated into two or more smaller bodies (Figure 4d). Similarly in *L. botrana* females, highly polyploid nuclei of fifth instar larvae showed a very tiny (or none) W-body (Figure 4j), while branched nuclei of adult moths showed several smaller sex chromatin bodies (Figure 4k). The W-body fragmentation indicates that some parts of the W chromosomes in *G. molesta* and *L. botrana* are composed of transcriptionally active euchromatin, which might affect the W-body formation as shown in structural mutants of the W chromosome in the flour moth, *Ephestia kuehniella* [45], [46].

**Figure 5:**
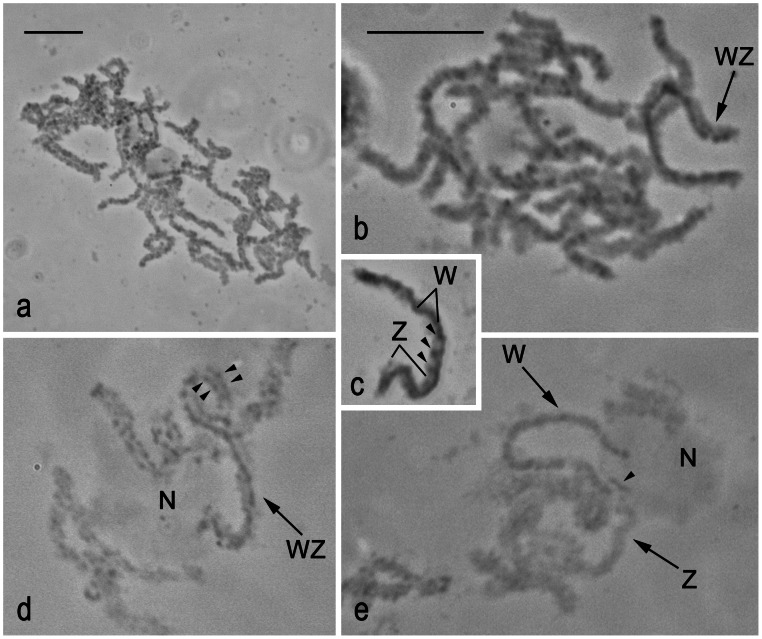
Identification of WZ bivalents in orcein-stained preparations of pachytene oocytes of three tortricid species. **a** – pachytene complement of *Grapholita molesta*; the WZ bivalent is indistinguishable. **b** – incomplete pachytene nucleus of *Lobesia botrana*; note a WZ bivalent (arrow) identified according to W-chromosome heterochromatin. **c** – a WZ bivalent of *L. botrana*; note the deeply stained W-chromosome thread while the Z-chromosome thread shows a chromomere pattern (see arrowheads pointing to deeply stained chromatin beads). **d** – a part of pachytene nucleus of *Eupoecilia ambiguella* with a WZ bivalent (arrow) anchored by one end in the nucleolus (N); note that most of the W chromosome is formed by a continuous thread except the end opposite to the nucleolar end, which shows a chromomere pattern similar to the Z chromosome (arrowheads). **e** – a part of zygotene/early pachytene nucleus of *E. ambiguella* with not yet paired sex chromosomes; note W and Z univalents (arrows) anchored by one end in the nucleolus (N); also note a deeply stained Z-end (arrowhead) inbuilt in the nucleolus. Bar = 10 µm; **b–e** have the same scale.

To identify the WZ bivalent we first examined spread preparations of pachytene oocytes stained with lactic acetic orcein in three tortricid species, *G. molesta*, *L. botrana*, and *E. ambiguella*. This research was not done in *G. funebrana*, as we did not find an optimal stage of larvae in the field-collected samples of this species. In *G. molesta*, most pachytene bivalents showed an indistinctive pattern of chromomeres, and no bivalent was discernible from autosomal bivalents by W heterochromatin (Figure 5a). Whereas in *L. botrana*, a WZ bivalent was easily identified according to the deeply stained thread of the W chromosome (Figure 5b), clearly differentiated from the Z chromosome that was weakly stained except for several conspicuous chromomeres (Figure 5c). The WZ bivalent was also easily discernible in pachytene complements of *E. ambiguella*. One end of the bivalent was associated with the large nucleolus (Figure 5d). In some nuclei with not yet paired sex chromosomes, both the W and Z chromosome univalents were anchored in the nucleolus (Figure 5e). This finding is consistent with the results of rDNA-FISH, which localized rDNA clusters to the ends of both the W and Z chromosomes (see Figure 2h). The W chromosome of *E. ambiguella* was composed of a continuous thread of heterochromatin except for the end distal to the nucleolus, which showed a chromomere pattern similar to the Z chromosome (Figure 5d).

**Figure 6:**
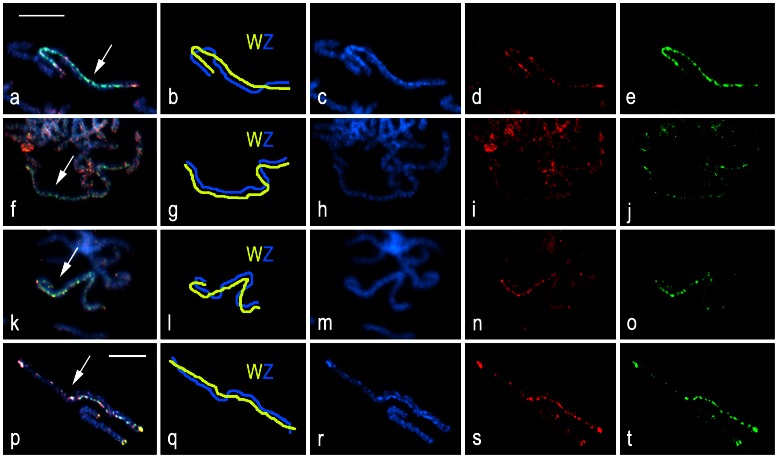
Comparative genomic hybridization (CGH) in pachytene oocytes of *Cydia pomonella* (a-e), *Grapholita molesta* (f-j), *Lobesia botrana* (k-o), and *Eupoecilia ambiguella* (p-t). Chromosomes were counterstained with DAPI (blue); female-derived genomic probes were labeled with fluorescein-12-dUTP (green), male-derived genomic probes with Cy3-dUTP (red). Figures **a–e**, **f–j**, **k–o**, and **p–t** show a detailed analysis of individual WZ bivalents: **a**, **f**, **k**, **p** – merged image of both probes including counterstaining; **b**, **g**, **l**, **q** – schematic interpretation of WZ bivalents; **c**, **h**, **m**, **r** – DAPI image; **d**, **i**, **n**, **s** – male genomic probe; **e**, **j, o**, **t** – female genomic probe. Arrows indicate WZ bivalents. Bar = 10 µm; **a–o** and **p–t** have the same scale.

Molecular differentiation of the W and Z chromosomes was examined using CGH. In pachytene oocytes of each tortricid species (except *G. funebrana*; see above), CGH identified the WZ bivalent by strong binding of both the female-derived and male-derived genomic probes to the W chromosome, with slight preference for the female probe (Figures 6a–t). However, we found considerable between-species differences in the distribution of hybridization signals. In *C. pomonella*, which was used as a control, both probes strongly bound to the W thread of the WZ bivalent, except for short terminal segments at both ends that were less labeled with the female probe (Figure 6a, d, e). The W chromosome was also highlighted with DAPI (Figure 6c). A similar CGH pattern was reported by [32]. The W chromosome of *G. molesta* was also highlighted by both probes, but hybridization signals were much weaker and scattered along the entire W length, consistently with indistinctive staining pattern of DAPI (Figure 6f, h–j). In *L. botrana*, the W chromosome was decorated with strong but scattered hybridization signals of both probes in more than half of the WZ bivalent; however, in the remaining part the W chromosome was almost indistinguishable from the Z chromosome (Figure 6k, m–o). Similarly, the W chromosome of *E. ambiguella* showed a continuous pattern of strong hybridization signals of both probes in more than half of the WZ bivalent, while the remaining part showed only a few spots evenly highlighted with both probes (Figure 6p, r–t). Interestingly, the pattern of hybridization signals was fully coincident with the heterochromatic segments highlighted with DAPI (Figure 6r).

## Discussion

Our study confirmed highly conserved basic features of tortricid karyotypes. All three Olethreutinae species (*G. funebrana*, *G. molesta*, and *L. botrana*) have a haploid chromosome number of n = 28 like our reference species, the codling moth (*C. pomonella*). The n = 28 seems to be a modal number in this subfamily. So far, it has been found in 11 out of 18 species examined, including all three species of the tribe Grapholitini, six out of nine species of Eucosmini, and two out of three species of Olethreutini but not in any of three Bactrini species (Table 1). However, the latter three species of the genus *Bactra* represent a special case with considerably reduced chromosome numbers, most likely due to chromosome fusions, and with multiple sex chromosomes in two of them [30]. Conversely, the subfamily Tortricinae exhibits much greater karyotype stability with a clear modal chromosome number of n = 30 that has been reported for 25 out of 26 species from four tribes, including *E. ambiguella* (Cochylini) examined in this study, two species of Sparganothini, three species of Tortricini, and 19 out of 20 species of Archipini (Table 1). The only exception found in Totricinae is the rustic tortrix, *Clepsis senecionana*, with n = 29 [24].

**Table 1:** Karyotype numbers (2n) in Tortricidae.

Tribe	Species^a^	2n	Reference(s)
Subfamily Tortricinae			
Archipini	*Adoxophyles orana*	60	[5], [23]
	*Aphelia paleana*	60	[24]
	*Archips breviplicanus*	60	[5], [23]
	*Archips cerasivorana* b	60	[5], [23], [25]
	*Archips crataegana*	60	[27]
	*Archips fervidana*	60	[25]
	*Archips fuscocupreanus*	60	[5], [23]
	*Choristoneura biennis*	60	[25], [28]
	*Choristoneura conflictana*	60	[25]
	*Choristoneura fumiferana*	60	[5], [25], [28]
	*Choristoneura lambertiana*	60	[28]
	*Choristoneura occidentalis*	60	[25], [28]
	*Choristoneura orae*	60	[28]
	*Choristoneura pinus*	60	[5], [25], [28]
	*Choristoneura retiniana*	60	[28]
	*Clepsis senecionana*	58	[24]
	*Homona coffearia* c	60	[5]
	*Homona magnanima*	60	[5], [23]
	*Lozotaenia forsterana*	60	[24]
	*Pandemis heparana*	60	[5], [23]
Cochylini	*Eupoecilia ambiguella*	60	this study
Sparganothini	*Cenopis penitana*	60	[25]
	*Sparganothis directana*	60	[25]
Tortricini	*Acleris forsskaleana*	60	[24]
	*Acleris variana*	60	[25]
	*Tortrix viridana*	60	[26]
Subfamily Olethreutinae			
Bactrini	*Bactra furfurana*	33/32^i^	[30]
	*Bactra lacteana*	31/30^i^	[30]
	*Bactra robustana*	46	[30]
Eucosmini	*Blastesthia tessulatana* d	56	[26]
	*Epinotia radicana* e	58	[25]
	*Epinotia solandriana*	56	[25]
	*Gypsonoma haimbachiana*	50	[25]
	*Retinia albicapitana* f	54	[25]
	*Rhyacionia buoliana*	56	[25]
	*Zeiraphera canadensis*	56	[25]
	*Zeiraphera fortunana*	56	[25]
	*Zeiraphera griseana* g	56	[31]
Grapholitini	*Cydia pomonella*	56	[26], [32]
	*Grapholita funebrana*	56	this study
	*Grapholita molesta*	56	this study
Olethreutini	*Lobesia botrana*	56	this study
	*Phiaris mori*	44	[29]
	*Pseudosciaphila duplex* h	56	[25]

a Species names are used according to [19];

b syn. *Choristoneura cerasivorana;*

c syn. *Homona menciana;*

d syn. *Pseudococcyx tessulatana;*

e syn. *Epinotia (Griselda) radicana;*

f syn. *Petrova albicapitana;*

g *Zeiraphera diniana;*

h *Sciaphila duplex;*

i species with multiple sex chromosomes W_1_W_2_Z/ZZ (female/male).

Another conserved feature of the tortricid karyotype is a large pair of the sex chromosomes that was found in the codling moth [32], [40], all four species examined in this study (except the middle-sized W chromosome in *G. funebrana*), and also found but not shown in the larch budmoth, *Zeiraphera griseana*, syn. *Z. diniana* (Guenée) (Olethreutinae; Eucosmini), with n = 28 ([31]; F. Marec, unpublished data on WZ bivalent). A similar large chromosome pair, supposed to be a pair of sex chromosomes, was also reported for almost each karyotyped tortricid species with modal or close to modal chromosome numbers [23]–[28]. Provided that the ancestral number in Lepidoptera is n = 31 and that a typical karyotype shows a gradual decrease in chromosome size [6], [7], the invariable presence of large sex chromosomes suggests that a common ancestor of Tortricinae and Olethreutinae had a reduced karyotype with n = 30 as a result of a sex chromosome-autosome fusion. This chromosome number has been conserved in most Tortricinae species, but was further reduced to n = 28 in Olethreutinae, most probably by two fusion events involving autosomes. Then subsequent multiple chromosome fusions within Olethreutinae resulted in derived karyotypes with much lower chromosome numbers as found in the genus *Bactra* [30]. This scenario of karyotype evolution is consistent with recent evidence obtained in the codling moth, *L. botrana*, and *E. ambiguella*, showing that the tortricid Z chromosome is a neo-Z that has arisen by fusion between an ancestral Z chromosome and an autosome corresponding to chromosome 15 in the silkworm, *B. mori*, and that this happened in a common ancestor of the main tortricid subfamilies, Olethreutinae and Tortricinae [41].

To further explore physical characteristics of tortricid karyotypes we mapped chromosomal distribution of two multigene families, major rDNA (i.e. 18S, 5.8S and 28S rRNA gene clusters) and H3 histone genes. Interestingly, the tortricid species examined showed a single rDNA cluster (except codling moth) and a single cluster of H3 genes (including codling moth) per haploid genome. Each rDNA or histone gene site was associated with a small block of DAPI-positive heterochromatin, not seen in two rDNA sites of the codling moth [32]. However, distribution patterns of the two chromosome markers greatly differed. A similar, nearly central position of the H3 gene cluster in a middle-sized autosome with a slight exception for *L. botrana*, where the cluster was positioned more eccentrically, suggests a conservative pattern of the H3 histone gene location in tortricids. The finding is consistent with a highly conservative number (i.e. one per haploid genome) and chromosomal location of H3 gene clusters reported recently in other insects, such as Acrididae grasshoppers [47] and Scarabaeinae beetles [48]. On the contrary, major rDNAs showed a variable chromosome location in tortricids, irrespective of evolutionary relationships [20], [21]. This is particularly obvious in closely related species, *G. molesta* and *G. funebrana* with a single rDNA cluster in a terminal position and an interstitial position, respectively, which contrasts with two terminal rDNA clusters at opposite ends of the NOR-chromosome in *C. pomonella* [32]. Moreover, a different rDNA location was also found in the other two species, specifically a subterminal rDNA cluster in an autosome of *L. botrana* and a terminal rDNA cluster in both the W and Z sex chromosomes of *E. ambiguella*. The high rDNA mobility in tortricids supports the concept of dynamic evolution of rDNA in Lepidoptera [35] and adds to growing evidence for the recognition of mobility as a common property of the major rDNAs, substantiated in extensive surveys of different organisms including insects [48]–[50].

There are two interesting features in the distribution of the major rDNAs in tortricids. One of them is the NOR-autosome bearing two terminal rDNA clusters in *C. pomonella*. Fuková et al. [32] hypothesized that the curious NOR-autosome might have arisen through fusion of two ancestral NOR-chromosomes by their non-rDNA ends. However, the hypothesis lacks a support in our results. On the contrary, the single rDNA cluster in the other tortricids examined suggests that a common ancestor of Olethreutinae and Tortricinae had a single NOR-chromosome pair. Hence the two terminal rDNA sites in *C. pomonella* are more likely result of rDNA expansion, for example, by ectopic recombination that has been proposed as a primary motive force of rDNA dynamics in Lepidoptera [35]. Since the family Tortricidae is the most basal lineage examined for NOR distribution so far (for Lepidoptera phylogeny, see [14], [51]), we can hypothesize that a single NOR was present also in the ancestral lepidopteran karyotype with n = 31. The other interesting feature is the W- and Z-location of rDNA in *E. ambiguella*, which has so far been only reported in the butterfly *Bicyclus anynana* [52] and the tussock moth *Orgyia thyellina*, though in the latter species the NOR was located in the originally autosomal part of the neo-sex chromosomes [53]. However, the sex-chromosome location of rDNA in other insects seems to be rather common as shown, for example, in tiger beetles of the genus Cicindela [54], bushcrickets of the genus *Odontura* [55], and Triatominae bugs [56]. In dipteran insects, the association of rDNA with sex chromosomes even seems to be an ancestral character for the whole order [57]–[60]. The rare occurrence on the sex chromosomes in Lepidoptera suggests that the sex-linkage of rDNA is not favorable, possibly due to the inactivation of the W chromosome in somatic nuclei of females [44].

The sex chromosomes of tortricids examined here showed some common features in addition to their large size. Similar to codling moth, the W chromosomes were largely composed of heterochromatin, and in CGH experiments they were differentiated by both the female and male genomic DNA probes, with slight preference for the female probes. These results suggest preponderance of common repetitive sequences and transposons and a low amount of W-specific sequences on the W chromosome (cf. [32], [37], [40]). However, a detailed analysis carried out in this study revealed considerable between-species differences in the formation of W chromatin bodies in the highly polyploid somatic nuclei of females (see Results), in the level of W-chromosome heterochromatinization, and in the pattern of molecular differentiation of the W and Z chromosomes. Only the codling moth W chromosome showed a conspicuous heterochromatinization and uniform CGH pattern along the entire W thread of the pachytene WZ bivalents ([32]; this study), unlike the indistinctive and scattered pattern of the W chromosome in the closely related *G. molesta*.

On the contrary, the CGH patterns in *L. botrana* and *E. ambiguella* suggest that their W chromosomes are composed of two parts, the highly differentiated and poorly differentiated parts. The latter finding strongly suggests that not only the Z chromosome (see above) but also the tortricid W chromosome had originated by fusion between an ancestral W chromosome (the highly differentiated part) and an autosome (the weakly differentiated part), most probably also corresponding to the *B. mori* chromosome 15 (see [41]). Following the W chromosome-autosome fusion event, the complete absence of meiotic recombination in lepidopteran females resulted in independent molecular degeneration of the autosomal part of the neo-W chromosome in different lineages of tortricids. The resulting molecular divergence could be then responsible for the observed between-species differences in the structure and composition of the tortricid W chromosomes.

In conclusion, our study confirmed conserved karyotypes of tortricids in terms of chromosome numbers, n = 30 in Tortricinae and n = 28 in Olethreutinae, and the large pair of the WZ sex chromosomes. However, differences in the molecular differentiation of the W chromosomes and in the pattern of rDNA distribution suggest a divergence in the internal architecture of tortricid karyotypes. In the codling moth, there is an interest to develop genetic sexing strains with the aim to increase the efficiency of the pest control using SIT [42]. For the creation of genetic sexing strains it has been proposed to insert a dominant conditional lethal mutation into the maternally inherited W chromosome [61]. A similarity between tortricid karyotypes along with the intimate knowledge of their sex chromosomes ([41]; this study) supports the application of technologies developed for the codling moth in other tortricid pests.
